# The Roles of Immunoregulatory Networks in Severe Drug Hypersensitivity

**DOI:** 10.3389/fimmu.2021.597761

**Published:** 2021-02-26

**Authors:** Yun-Shiuan Olivia Hsu, Kun-Lin Lu, Yun Fu, Chuang-Wei Wang, Chun-Wei Lu, Yu-Fen Lin, Wen-Cheng Chang, Kun-Yun Yeh, Shuen-Iu Hung, Wen-Hung Chung, Chun-Bing Chen

**Affiliations:** ^1^ Department of Medical Education, Chang Gung Memorial Hospital, Linkou, Taiwan; ^2^ College of Medicine, Chang Gung University, Taoyuan, Taiwan; ^3^ Department of Dermatology, Drug Hypersensitivity Clinical and Research Center, Chang Gung Memorial Hospital, Linkou, Taiwan; ^4^ Department of Dermatology, Drug Hypersensitivity Clinical and Research Center, Chang Gung Memorial Hospital, Taipei, Taiwan; ^5^ Department of Dermatology, Drug Hypersensitivity Clinical and Research Center, Chang Gung Memorial Hospital, Keelung, Taiwan; ^6^ Cancer Vaccine and Immune Cell Therapy Core Laboratory, Chang Gung Memorial Hospital, Chang Gung Immunology Consortium, Linkou, Taiwan; ^7^ Immune-Oncology Center of Excellence, Chang Gung Memorial Hospital, Linkou, Taiwan; ^8^ Department of Nursing, Chang Gung Memorial Hospital, Linkou, Taiwan; ^9^ Division of Hematology-Oncology, Department of Internal Medicine, Chang Gung Memorial Hospital at Linkou, Chang Gung University College of Medicine, Taoyuan, Taiwan; ^10^ Division of Hematology-Oncology, Department of Internal Medicine, Chang Gung Memorial Hospital, Keelung, Taiwan; ^11^ Department of Dermatology, Chang Gung Hospital, Xiamen, China; ^12^ Department of Dermatology, Beijing Tsinghua Chang Gung Hospital, School of Clinical Medicine, Tsinghua University, Beijing, China; ^13^ Department of Dermatology, Ruijin Hospital, School of Medicine, Shanghai Jiaotong University, Shanghai, China; ^14^ Genomic Medicine Core Laboratory, Chang Gung Memorial Hospital, Linkou, Taiwan; ^15^ College of Medicine, Graduate Institute of Clinical Medical Sciences, Chang Gung University, Taoyuan, Taiwan

**Keywords:** contact dermatitis, delayed type hypersensitivity, drug reaction with eosinophilia and systemic symptoms, cosignaling pathways, immune checkpoints, regulatory T cells, Stevens-Johnson Syndrome, toxic epidermal necrolysis

## Abstract

The immunomodulatory effects of regulatory T cells (Tregs) and co-signaling receptors have gained much attention, as they help balance immunogenic and immunotolerant responses that may be disrupted in autoimmune and infectious diseases. Drug hypersensitivity has a myriad of manifestations, which ranges from the mild maculopapular exanthema to the severe Stevens-Johnson syndrome (SJS), toxic epidermal necrolysis (TEN), and drug reaction with eosinophilia and systemic symptoms/drug-induced hypersensitivity syndrome (DRESS/DIHS). While studies have identified high-risk human leukocyte antigen (HLA) allotypes, the presence of the HLA allotype at risk is not sufficient to elicit drug hypersensitivity. Recent studies have suggested that insufficient regulation by Tregs may play a role in severe hypersensitivity reactions. Furthermore, immune checkpoint inhibitors, such as anti-CTLA-4 or anti-PD-1, in cancer treatment also induce hypersensitivity reactions including SJS/TEN and DRESS/DIHS. Taken together, mechanisms involving both Tregs as well as coinhibitory and costimulatory receptors may be crucial in the pathogenesis of drug hypersensitivity. In this review, we summarize the currently implicated roles of co-signaling receptors and Tregs in delayed-type drug hypersensitivity in the hope of identifying potential pharmacologic targets.

## Introduction

Hypersensitivity reactions refer to undesirable immune reactions that are exaggerated or inappropriate against an antigen or allergen. Generally, hypersensitivity reactions are classified into four types, which differ in pathogenesis, clinical manifestation, and prognosis (1). And when it comes to the treatment of these allergic reactions, type IV hypersensitivity remains the most challenging. Type IV hypersensitivity reaction is also called delayed-type hypersensitivity (DTH) because it reaches its peak 48 to 72 h after exposure to drugs, allergens, or toxins (2). DTH is mediated by direct cytotoxicity of CD8+ T cells or by the release of cytokines from CD4+ cells, which act through antigen-presenting cells (APC) such as macrophages and dendritic cells (DCs) to stimulate chronic inflammatory reactions. Therefore, unlike other types of hypersensitivity reactions that are antibody-dependent, DTH is majorly a T cell-mediated response, which accounts for a variety of drug eruptions including severe cutaneous adverse reactions (SCARs), and contact hypersensitivity (CHS) or allergic contact dermatitis (ACD) (3–5).

SCARs include syndromes such as Stevens-Johnson syndrome (SJS), toxic epidermal necrolysis (TEN), and drug reaction with eosinophilia and systemic symptoms/drug-induced hypersensitivity syndrome (DRESS/DIHS) (5). SJS/TEN is a rare but life-threatening drug-induced cutaneous reaction characterized by extensive necrosis and detachment of epidermis and mucosal epithelium (6). SJS and TEN share the same clinical pattern, histopathologic features, and mechanisms; though SJS is defined by the involvement of less than 10% of total body surface area (TBSA) whereas TEN indicates detachment of more than 30% of TBSA (7, 8). On the other hand, DRESS/DIHS is also a potentially fatal drug-induced systemic hypersensitivity syndrome which mainly causes skin and internal organ damage (9–11). Patients with uncontrolled DRESS/DIHS or complications such as cytomegalovirus (CMV) reactivation are at high risk of death (12). Aside from drug eruptions, CHS or ACD is also an inflammatory disease of the skin that is primarily T cell-mediated (13). ACD results from exposure and sensitization of hosts to specific allergens, followed by subsequent exposure that initiates an inflammatory response, causing skin damage and inflammation (3).

Since the treatment of the DTH entity remains challenging, there is a need for physicians to look into the detailed interactions between immune cells and explore novel therapeutic targets (14–17). Fortunately, current evidence has demonstrated that both the dysregulations of regulatory T cells (Tregs) and the dysfunction of certain co-signaling axes are associated with the development of cutaneous inflammatory disease (18, 19). In this review, we summarize the established pathogenesis of clinically-relevant DTHs then focus on currently suggested concepts regarding how Tregs and various costimulatory and coinhibitory receptors regulate different components and cytokines in DTH in the hope of clarifying potential targets for future treatment.

## Current Models of Pathogenesis in Drug Hypersensitivity and Beyond

Delayed-type hypersensitivities ranging from CHS to SCARs remain as major therapeutic challenges in clinical scenarios (20, 21). Upon exposure to a certain drug, not only does the drug itself but also its metabolites and drug-modified peptides are often present systemically (22, 23). Importantly, some of them could bind to human leukocyte antigen (HLA) and activate T cells, potentiating hypersensitivity (24, 25). Previous studies on drug hypersensitivity had identified several models explaining the pairwise associations between specific HLA class I alleles and the susceptibility to certain drug hypersensitivity, namely the hapten/pro-hapten model, the pharmacologic interaction model, and the altered peptide repertoire model, which have been thoroughly reviewed (26, 27).

After activating certain drug-specific T lymphocytes, cytotoxic molecules ranging from granulysin to granzyme B (GzmB) and a variety of proinflammatory cytokines including but not limited to tumor necrosis factor (TNF), interferon (IFN)-γ, and interleukin (IL)-2, are released, which collectively lead to unwanted inflammatory damages (28–31). In addition to cytotoxic T cells, activated natural killer (NK) cells, T helper 17 cells (Th17), and APCs also contribute to the release of IL-5, IL-6, IL-12, IL-15, IL-17, IL-18, further exacerbating the extensive collateral damages and result in SJS/TEN (27, 32). On the other hand, when the activation of certain drug-specific T helper 2 (Th2) lymphocytes occurs with the reactivation of human herpes virus (HHV)-6, HHV-7, Epstein–Barr virus (EBV), or CMV, these T cells are thought to further release IL-4, IL-5, and IL-13 which work together with eotaxin, thymus activation-regulated chemokine (TARC), and pro-inflammatory cytokines such as IFN-γ, TNF, IL-6, and IL-15, promoting systemic inflammation with eosinophilia and resulting in DRESS/DIHS (15, 33–36). Thanks to the high negative predictive values of genetic screenings for HLA, physicians could achieve early prevention of these fatal drug hypersensitivities prior to drug use (37–39). However, there are only a few HLA-typing (e.g., HLA-B*57:01 for abacavir, HLA-B*58:01 for allopurinol, and HLA-B*15:02 for carbamazepine) that have been recommended to prevent drug hypersensitivity in practice, and more attention is warranted to explore the applicability of this strategy in other dozens of drug-related DTH.

Besides the HLA alleles, the T cell receptor (TCR) repertoire also plays an important role in the pathogenesis of SCAR. Recently, we identified a public TCR from carbamazepine-induced SJS/TEN patients of Asians and Europeans. This observation may explain why patients of different ethnicities with different HLA alleles develop the same hypersensitivity. This public TCR shows drug-specificity and phenotype-specificity in an HLA allele-specific (HLA-B*15:02-favored) manner (40). Intriguingly, despite the presence of the culprit molecules systemically as well as the corresponding HLA type and TCR, many people remain unaffected while others develop severe drug hypersensitivity, implying that binding to drug-specific T cells per se is not sufficient to be pathogenic. The missing pieces to explain the unpredictability here could partially be attributed to both the activity of the Treg and the collateral co-signaling axes that largely determine the activation and effector functions of T cells, which had been extensively explored in fields targeting tumors as well as autoimmune diseases (41, 42).

**Figure 1:**
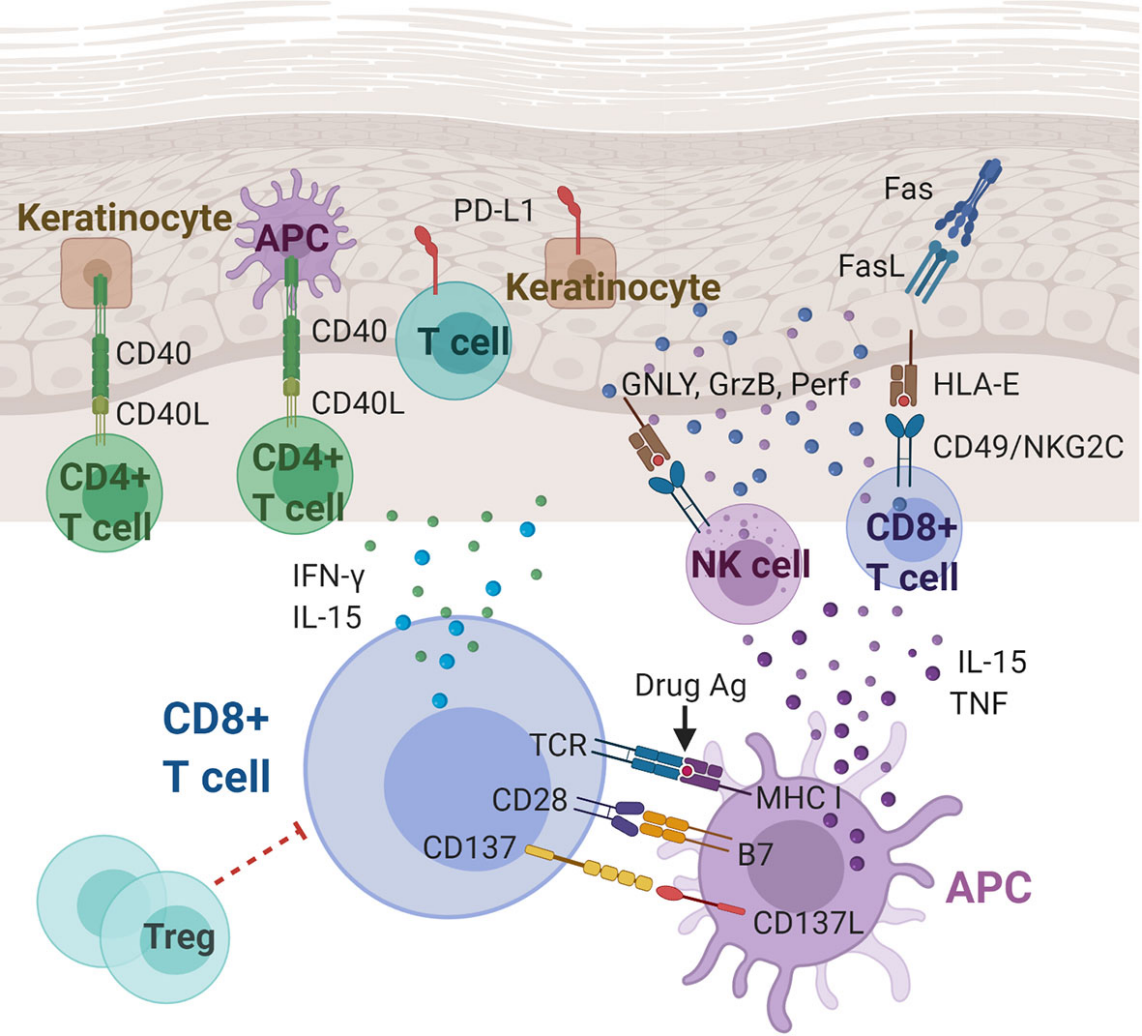
Immunoregulatory molecules and cytokines implicated in Stevens-Johnson syndrome/toxic epidermal necrolysis (SJS/TEN). CD8+ cytotoxic T lymphocytes (CTLs) play a central role in the pathogenesis of SJS/TEN. Cytokines (including IL-15 and TNF) and costimulatory molecules of APCs can stimulate the CTLs, which in turn produce cytokines, including IFN-γ and IL-15. CTL and NK cell degranulation that induce keratinocyte apoptosis may be mediated, at least partially, by the interaction between CD49/NKG2C and HLA-E. Other players in SJS/TEN include Fas/FasL interactions, T cells and keratinocytes expressing PD-L1, and CD40/CD40L interactions at the dermal-epidermal junction. Ag, antigen; APC, antigen presenting cell; CD40(L), cluster of differentiation 40 (ligand); FasL, Fas ligand; GNLY, granulysin; GzmB, granzyme B; HLA-E, HLA class I histocompatibility antigen, alpha chain E; IFN-γ, interferon gamma; IL-15, Interleukin 15; MHC, major histocompatibility complex; NK cell, natural killer cell; Perf, perforin; PD-L1, programmed death ligand-1; TCR, T cell receptor; TNF, tumor necrosis factor; Treg: regulatory T cell.

**Figure 2:**
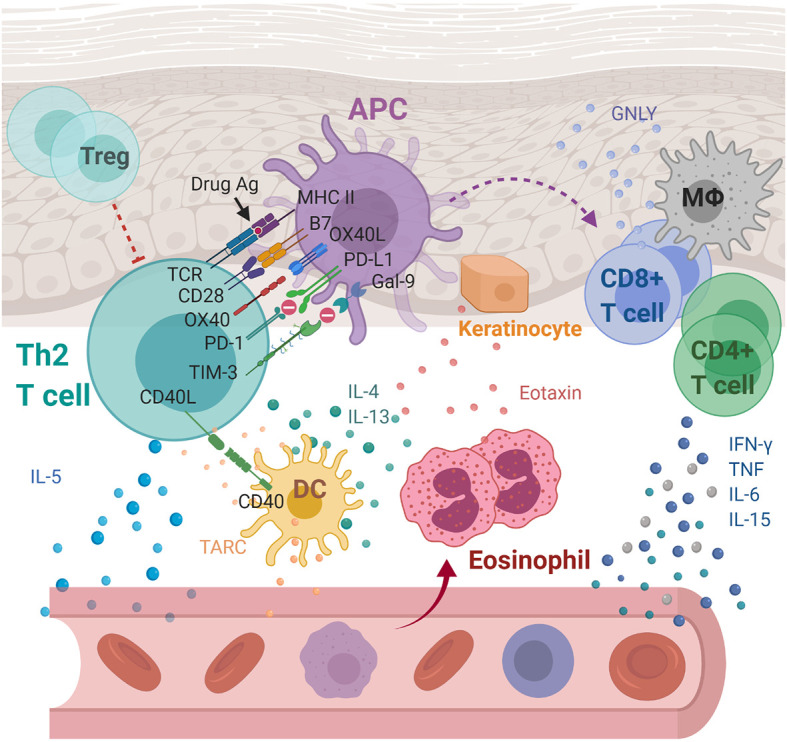
Immunoregulatory molecules and cytokines implicated in drug reaction with eosinophilia and systemic symptoms/drug-induced hypersensitivity syndrome (DRESS/DIHS) and maculopapular exanthema (MPE). Through cell-cell interaction, APCs are thought to activate drug-specific T cells with the aid of OX40 costimulation which prevent T cells from being inhibited by Tregs. Activated Th2 secretes cytokines including IL-4, IL-5, and IL-13, inducing eosinophilia. In addition, eotaxin and TARC produced by keratinocytes and DCs, respectively, promote the local accumulation of harmful eosinophils. Together with the elevated levels of pro-inflammatory cytokines, including IFN-γ, TNF, IL-6, and IL-15, they cause systemic inflammation characterized as DRESS/DIHS. In the case of MPE, the augmented immune responses through the CD40 axis along with the compromised inhibitory mechanisms of both PD-1 and TIM-3 axes were further identified. APC, antigen presenting cell; DC, dendritic cells; Gal-9, galectin-9; GNLY, granulysin; IFN-γ, interferon gamma; IL-4, interleukin 4; IL-5, interleukin 5; IL-6, interleukin 6; IL-13, interleukin 13; IL-15, interleukin 15; MΦ, macrophage; MHC, major histocompatibility complex; PD-1, programmed cell death protein 1; PD-L1, programmed death ligand-1; TARC, thymus activation-regulated chemokine; TCR, T cell receptor; Th2, T helper 2 cell; TIM-3, T-cell immunoglobulin mucin-3; TNF, tumor necrosis factor; Treg, Regulatory T cell.

**Figure 3:**
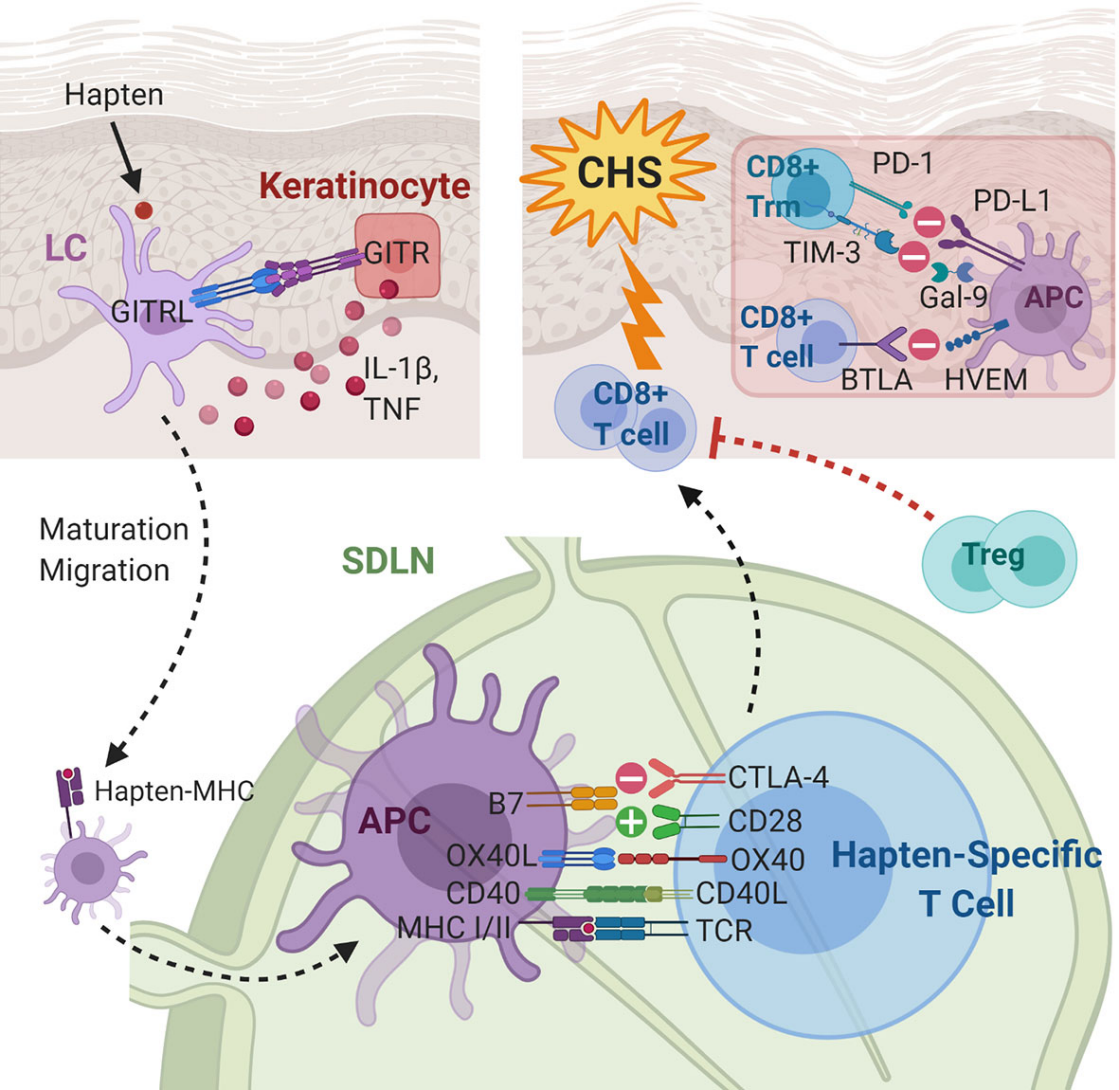
Immunoregulatory molecules and cytokines implicated in contact hypersensitivity (CHS). During sensitization, the skin-penetrating hapten is processed by Langerhans cells (LCs) or dermal dendritic cells. GITR-GTRL interactions between keratinocytes and LCs enhance keratinocyte secretion of cytokines, which aid maturation and migration of LCs to the skin-draining lymph node. In the SDLN, in addition to hapten presentation, several costimulatory and coinhibitory interactions, e.g., CD40-CD40L, OX40L-OX40, B7-CD28, and B7-CTLA-4, between the APC and the naïve T cell take place. The hapten-specific effector T cells, predominantly CD8+ cytotoxic T cells, infiltrate the skin and elicit the CHS response upon hapten re-exposure. The presence of Tregs and several coinhibitory receptors on CD8+ T cells and CD8+ Trm cells are associated with decreased CHS response. APC, antigen presenting cell; BTLA, B- and T-lymphocyte attenuator; CD40L, CD40 ligand; CTLA-4, cytotoxic T-lymphocyte-associated antigen 4; Gal-9, galectin-9; GITR(L), glucocorticoid induced TNF receptor (ligand); HEVM, herpes virus entry mediator; IL-1β, interleukin-1 beta; LC, Langerhans cell; MHC, major histocompatibility complex; OX40L, OX40 ligand; PD-1, programmed cell death protein 1; PD-L1, programmed death ligand-1; SDLN, skin-draining lymph nodes; TCR, T cell receptor; TIM-3, T-cell immunoglobulin mucin-3; TNF, tumor necrosis factor; Treg, regulatory T cell; Trm, tissue-resident memory T cell.

Besides the pathophysiology of different drug hypersensitivity reactions identified previously, insufficient inhibitory mechanisms and/or enhanced costimulatory signals to T cells have also been gaining attention in the pathomechanism of SJS/TEN and DRESS/DIHS as outlined in **Figures 1** and **2**, respectively. Furthermore, since previous research on DTH has largely focused on CHS, reviewing costimulatory/coinhibitory signaling in CHS may help guide future drug hypersensitivity research; thus, the co-signaling pathways suggested in the pathogenesis of CHS are also depicted (**Figure 3**). Clarifying the immunological crosstalk may open the therapeutic window to further prevent and control potentially fatal drug hypersensitivity.

## Costimulatory Receptors

Different HLA alleles represent important risk factors for drug hypersensitivity by specific drugs in patients of specific genetic ancestry. However, evidence thus far seems to suggest that the commonly discussed variations of the HLA genotypes are not sufficient to fully predict susceptibility to DTH (43–47). It is known that alongside the TCR/peptide-MHC interaction, “signal 2” is crucial for proper activation of T cells by antigen-presenting cells. Aside from the prototypic costimulatory receptor, CD28, that is involved in this process (48), various costimulatory receptors have also been demonstrated to facilitate the immune responses in autoimmune diseases, cancer, and infectious diseases (49–51). Costimulatory receptors implicated in DTH are summarized in **Table 1**.

**Table 1:** Costimulatory molecules involved in delayed-type hypersensitivity.

Costimulatory molecules	Implicated cells in DTH	Ligand/receptors	Ligand/receptors expression	Types of DTH	Study designs
CD28	T cells (including Tregs)	CD80, CD86	APCs	SJS/TEN; CHS; abacavir hypersensitivity	Animal study (52–55)
OX40	Tregs, conventional T cells	OX40L	APCs	CHS; DRESS/DIHS	Case control (56, 57); case series (58); animal study (59, 60)
CD40	Activated T cells, APCs	CD40L	APCs, activated T cells	CHS; sulfamethoxazole hypersensitivity; SJS/TEN; amoxicillin/amoxicillin-clavulanic acid DTH; anti-epileptic-induced hypersensitivity	Case control (61, 62); case series (63); animal study (64–66); *in vitro* study (67)
CD226	T cells, APCs	CD155	APCs, T cells	CHS	Animal study (68); *in vitro* study (69)
CD137/4-1BB	Activated T cells	CD137L/4-1BBL	CD14+CD16+ Monocyte, APCs	SJS/TEN	Case series (70)
GITR	Epidermal keratinocytes, T cells (including Tregs)	GITRL	APCs	CHS	Animal study (71)
CD94/NKG2C	NK cells, Cytotoxic T cells	HLA-E	Keratinocytes	SJS/TEN	Case control (72)

DTH, delayed-type hypersensitivity; CD28, cluster of differentiation 28; APC, antigen-presenting cell; CHS, contact hypersensitivity; SJS/TEN, Stevens-Johnson syndrome/toxic epidermal necrolysis; OX40L, OX40 ligand; DRESS/DIHS, drug reaction with eosinophilia and systemic symptoms/drug-induced hypersensitivity syndrome; GITR(L), glucocorticoid induced TNF receptor (ligand); NK cells, natural killer cells; HLA-E HLA class I histocompatibility antigen, alpha chain E.

### CD28

The costimulatory molecules B7-1 (CD80) and B7-2 (CD86) on APCs interact with their cognate receptor CD28 on T cells to augment and sustain a T cell response (73). CD28 costimulation promotes T cell proliferation, cytokine production, and T cell survival (74). In a transgenic mouse model for HLA-B*57:01–linked abacavir hypersensitivity, CD80 on DCs are upregulated upon CD4+ T cell depletion (likely including Tregs), resulting in an adverse immune response (52, 53). To further assess the effect of blocking this pathway on DTH, mice with antibodies targeting CD28 or CD80 inhibited the accumulation of reactive CD8+PD-1+ T cells in the lymph nodes, dampening the immune response to abacavir. In addition, CHS to dinitrofluorobenzene (DNFB) and oxazolone is significantly decreased in CD28 −/− mutant mice (54). CD28 antagonist has also been suggested prevent Aldara-induced skin inflammation in non-human primates, as shown by the inhibition of T cell and macrophage infiltration at the epidermal interface (55). Since studies on the role of CD28 in DTH have largely been animal-based, future studies to investigate the role of CD28 in DTH in humans are warranted before assessing the applicability of CD28 manipulation for therapeutic purposes.

### OX40 (CD134)

The costimulatory receptor OX40 (also known as CD134) is a member of the TNF receptor superfamily (TNFRSF) that is expressed on both activated CD4+ and CD8+ T cells, neutrophils, and NK cells (75). OX40 has important costimulatory functions in the activation, survival, and expansion of both CD4+ and CD8+ T cells as demonstrated in animal models of autoimmune disease, infectious disease, and cancer (76). Additionally, activation of T cells by the OX40 ligand (OX40L) makes them less responsive to the inhibitory signals from Tregs (77). One study demonstrated that mice lacking OX40L exhibited an impaired CHS response, due to defects in T cell priming and cytokine production (59). In another murine model of CHS, OX40L-deficient mice displayed a significant reduction in both hapten-induced ear swelling and hapten-specific T cell response. Conversely, these responses were markedly increased in mice with OX40L overexpression (60). In a comparison between eight patients of acute stage DRESS/DIHS and seven healthy controls, OX40 was found to be significantly upregulated on CD4+ T cells of DRESS/DIHS patients (56). In their recent follow-up study of 12 DRESS/DIHS patients, Miyagawa et al. have found that OX40L was also preferentially expressed on their peripheral blood mononuclear cells (PBMCs). Moreover, the percentage of OX40-expressing CD4+ T cells correlated with parameters observed in Th2-type immune responses (57). Additionally, during slow desensitization of five patients with previous imatinib-induced DTH, including maculopapular exanthema (MPE) and DRESS/DIHS, decreased imatinib-induced CD4+CD25+OX40+ T-cell percentages were observed (58), suggesting that decreased OX40+ drug-specific T cells may be associated with immune tolerance. Since co-expression of OX40 and CD25 after 48 h upon antigen stimulation has been described as a marker for antigen-specific CD4+ T cells (78), this result hints at the important role of OX40 in the T cell hypersensitivity response. Since studies on OX40 have largely focused on CHS in mice and DRESS/DIHS in humans, a comprehensive understanding of the role of OX40/OX40L in DTH may require future investigation of other types of DTH and the interactions between OX40 and other competing players in the immune response. On another note, though the potential therapeutic application of the OX40–OX40L interaction has not been explored in DTH, it has already been considered in autoimmune and cancer treatments (79).

### CD40 and CD40L

The CD40/CD40 ligand (CD40L or CD154) costimulatory axis also belongs to the tumor necrosis factor receptor superfamily (TNFRSF) and amplifies the immune response and promotes inflammation. While both are expressed on the surface of a variety of cell types, CD40L expression mostly requires induction whereas CD40 expression is often constitutive. Cells expressing CD40L include, but are not limited to, activated T cells, DCs, NK cells, platelets, and non-immune cells. Similarly, CD40 is expressed on B cells, T cells, DCs, and many other cell types (80). An *in vitro* study of T-cell-mediated hypersensitivity to sulfamethoxazole (SMX) showed increased CD40 expression with DC surfaces exposed to SMX and its metabolite, nitroso SMX (67). CD40L blockade inhibited nitroso SMX-induced T cell activation in mice. Additionally, CD40 stimulation enhanced the drug-specific response. In mice, blockade of the CD40-CD40L pathway was associated with defective CHS response, possibly due to impaired migration of antigen-bearing DCs from the skin to draining lymph nodes, while injection of the agonist anti-CD40 monoclonal antibody “corrected” the CHS response (64, 65). However, CD40-CD40L interactions may not be mandatory for the development of the effector CD8+ or the regulatory CD4+ T cells during DNFB sensitization for CHS in mice (66). In order to study the role of CD40 in human diseases, the cellular infiltrates of the skin of eight patients with erythema multiforme and six with SJS/TEN had been analyzed. While CD40+ cells were similarly represented in erythema multiforme and SJS/TEN, CD40L+CD4+ T cells which may bind to CD40 on APCs were strongly represented in the perivascular and subjunctional dermis of SJS/TEN specimens (63). This study also revealed CD40, Fas, and Fas ligand expressions on keratinocytes in SJS/TEN patients. Recently, the potential of using CD40L to detect activated drug-specific T cells in DTH to antibiotics and anti-epileptics has been demonstrated in case control studies of small sample sizes, but its overall accuracy and application in SCARs remain to be further explored. For instance, among 14 patients clinically diagnosed with drug eruptions, only 8 of their causative drug-activated PBMCs were tested positive for CD40L (61, 62).

### CD226 and CD155

The costimulatory receptor CD226 (DNAM-1) is a glycoprotein expressed on the majority of NK cells, T cells, monocytes and platelets, and a subset of B lymphocytes. CD226 competes with the coinhibitory T cell immunoglobulin and ITIM domain (TIGIT) for the same ligands, mediating positive stimulatory signaling and inducing NK and T cell‐mediated cytotoxicity (81, 82). Ligands for CD226 include CD155 (poliovirus receptor, PVR) and CD112 (nectin-2 or poliovirus receptor-related 2, PVRL2), which are expressed on epithelial cells, endothelial cells, APCs, and tumor cells; thus, the interaction between CD226 and its ligands is more often discussed in the context of tumor immunity (83, 84). A previous *in vitro* study had suggested that CD226 on CD4+ naive T cells mediated an activating signal for T helper 1 cell (Th1)/Th17 differentiation as a result of ligation with CD155 (69). Somewhat surprisingly, one murine study has demonstrated that CD155-mediated signaling in CD4+ T cells triggered by the interaction with CD226 on APCs was involved in Th1 development and CHS (68). CD155, normally regarded as the “ligand” in this costimulatory couple, was suggested to serve as the “receptor” when present on T cells. In the same study, anti-CD155 monoclonal antibody administration was able to dampen the CHS response. Whether the same manner of CD226/CD155 interaction is involved in human CHS has yet to be demonstrated.

### CD137 (4-1BB)

Another costimulatory receptor 4-1BB (CD137) is expressed on a vast array of cell types within the hematopoietic system, including both activated CD4+ and CD8+ T cells, DCs, B cells, monocytes, NK cells, neutrophils, and mast cells (85). The 4-1BB ligand (4-1BBL or CD137L) is expressed on most leukocytes, including APCs, and some non-immune cells. Interestingly, 4-1BBL can also transmit signals into the APC on which it is expressed (reverse signaling) and strengthen type 1, cell-mediated immune responses (86–88). The ability to potentiate effector responses by 4-1BB signaling in activated T cells makes 4-1BB an appealing target for cancer immunotherapy (89, 90). In one study, CD14+CD16+ cells of the monocyte lineage co-expressing CD80, CD86, and CD137L were found in the skin of all 11 SJS/TEN patients while interaction with CD8+CD137+ cells was also demonstrated on immunostaining (70). The interaction between cells bearing CD137 and cells bearing CD137L was suggested to contribute to epidermal damage most probably by enhancing the cytotoxicity of CD8+ T cell. Definitive conclusions cannot be drawn regarding CD137’s role in DTH due to limited evidence; further supporting studies on this costimulatory receptor are still needed.

### Glucocorticoid-Induced TNF Receptor

The costimulatory receptor GITR (glucocorticoid-induced TNF receptor, CD357) is a member of the TNFRSF that is constitutively expressed at high levels on Tregs, at lower levels on conventional T cells and innate immune cells, and on non-immune cells, e.g., keratinocytes (91, 92). The GITR ligand (GITRL) is predominantly expressed on activated APCs and endothelial cells (91, 93). Some functions of GITR include lowering the threshold of CD8+ T cell activation by CD28 and abrogating Treg-mediated suppression of T cells (94, 95). Studies on agonistic GITR in the context of cancer immunotherapy similarly demonstrated its dual ability to enhance CD8+ T cell proliferation and cytokine production while dampening Treg suppressive function (96, 97). In the CHS mouse model, the anti-GITRL monoclonal antibody inhibited hapten-specific CD8+ T cell activation and CHS. However, Tregs and plasmacytoid DCs, known to express high levels of GITR and GITRL, respectively, were not the main players in GITRL-mediated CHS. Rather, their results suggested that the binding of GITRL on Langerhans cells to the GITR on epidermal keratinocytes induced the expression of proinflammatory cytokines by the keratinocytes and the migration of Langerhans cells to the draining lymph nodes to initiate the CHS response (71). Since similar results have not been reported by other studies, this mechanism in CHS mandates further investigation.

### CD94/NKG2C

CD94 expressed on NK cells can form heterodimers with NKG2A (CD94/NKG2A) or with NKG2C (CD94/NKG2C). Binding of the heterodimer CD94/NKG2C to its ligand, HLA‐E, delivers an activating signal in NK cells (98). Interestingly, CD94/NKG2C is also expressed by highly differentiated CD8^+^ effector T cells and co-stimulates TCR‐mediated cytotoxicity (99). Keratinocytes from affected skin in SJS/TEN express HLA-E, which sensitizes them to killing by CD94/NKG2C-expressing cytotoxic T lymphocytes (CTLs) and NK cells. Moreover, in a case control study of SJS/TEN patients, activated blister T and NK cells expressing CD94/NKG2C degranulate in response to HLA-E+ cells in an NKG2C-dependent manner (72). And the cytolytic granules secreted by CTLs and NK cells have been previously demonstrated to include perforin, GzmB, soluble Fas ligand, and granulysin, which are important for keratinocyte killing of patients with SJS/TEN (30). Thus, the interaction between CD94/NKG2C on CTLs and NK cells and HLA-E on keratinocytes seems to contribute to the pathogenesis of SJS/TEN.

Since costimulatory signals boost the activation of T cells and are suggested to play a role in drug hypersensitivity, developing targeting therapies against them may be a plausible strategy against SCARs. Contrariwise, when it comes to the immunological regulations that hold back unwanted immune responses which we would like to augment, it is indispensable to discuss Treg and its multifaceted immunosuppressive abilities that could largely change the landscape of drug hypersensitivity (100, 101).

## Regulatory T Cells

Activation of the immune system is crucial for fending off foreign pathogens or annihilating endogenous malignancies. However, autoimmune diseases and hypersensitivity reactions are important illustrations of exaggerated immune responses to self and non-self antigens, respectively, that can be life-threatening. Maintaining immunologic self-tolerance and control requires a special subset of CD4+ T cells, the Tregs (102, 103). Tregs regulate immune system through multiple mechanisms, including the release of inhibitory cytokines, the initiation of cytolysis, and the modulation of DC function (104). Regulation through checkpoint receptors cytotoxic T lymphocyte-associated antigen-4 (CTLA-4) and programmed cell death 1 (PD-1) are also pivotal to the Treg function. By modulating CD4+ T cells, CD8+ T cells, B cells, and DCs, Tregs play a crucial role in the homeostasis of the immune response.

The constitutively expressed forkhead box protein 3 (FOXP3) is the lineage-defining transcription factor indispensable for Treg development and suppressive function (105, 106). Mutations in FOXP3 renders the *scurfy* transgenic mice and patients with IPEX (immunodysregulation polyendocrinopathy enteropathy X-linked) syndrome susceptible to spontaneous inflammation in different tissues, including the skin, and traits characteristic of connective tissue diseases (107–110). Recently, mapping regulation of gene expression in Tregs has revealed dysregulation of key Treg pathways in immune diseases, including many that display skin inflammation, providing hints to future drug targets for treatment (111).

### The Relevance of Regulatory T Cells in Delayed-Type Hypersensitivity

More Tregs than non-Tregs accumulate in the skin during cutaneous immune response in mice. Furthermore, mRNA expression profiles of cytokines in Tregs contain significantly higher amounts of the inhibitory *Il10*, *Tgfb1*, and *Ctla4* transcripts (112). Depletion of FOXP3+ Tregs in the PBMCs of healthy human donors allows the detection of CD4+ and CD8+ T cell responses to drugs and haptenic chemical, suggesting Treg’s canonical suppressive role (113). Not surprisingly, the reintroduction of Tregs blocks drug activation of naïve T cells in a cell concentration-dependent manner (114). In fact, the dysfunction of Tregs does contribute to the pathogenesis of drug hypersensitivity in clinical scenarios. For instance, the incidence and severity of drug hypersensitivity were increased in human immunodeficiency virus (115)-infected patients (116–118), whose CD4 counts and CD4/CD8 ratios decreased dramatically (118). Moreover, it was also observed that cancer patients receiving anti-CTLA4 and/or anti-PD-1 immunotherapy suffered from more drug hypersensitivity adverse events (119).

#### Stevens-Johnson Syndrome/Toxic Epidermal Necrolysis

Treg dysfunction has been suggested in the pathogenesis of SJS/TEN. In mice, Tregs prevent experimentally induced epidermal injury mimicking TEN (120). In humans, non-responders to carbamazepine re-exposure have a statistically significant expansion of Tregs compared to responders (121). In skin lesions of HIV patients with TEN, the decrease in the number of skin-directed CD4+ cells and the increase in the CD8+/CD4+ cell ratio likely contribute to an increased risk of developing drug reactions because of the loss of skin-protective regulatory T cells (116, 122). In another patient using the PD-1 inhibitor nivolumab, sequential mRNA expression of FOXP3 and the CD4+/CD8+ ratio reached its nadir at the onset of TEN in peripheral blood cells (123). Additionally, the percentage of Tregs in peripheral blood continued to drop in the course of TEN (123). As expected, Treg-mediated suppression of drug-specific T-cells in humans is seen with the addition of Tregs in a concentration-dependent manner (124). A comparison of cell composition in the skin of patients with erythema multiforme and SJS/TEN shows that Tregs are less abundant in the latter (125). Qualitative deficits of Tregs also seem to contribute to the pathogenesis of SJS/TEN. Circulating Tregs of SJS/TEN patients display impaired suppressor function that can be restored after clinical resolution (126). Notably, our recent study has also found that the TNF-α antagonist, etanercept, successfully relieves SJS/TEN symptoms with an increase in frequencies of CD4+CD25+FOXP3+ Tregs after treatment (127).

#### Drug Reaction with Eosinophilia and Systemic Symptoms/Drug-Induced Hypersensitivity Syndrome

Patients with DRESS/DIHS at the acute stage show significantly increased frequencies of Tregs in total CD4+ T cells compared with healthy controls in blood, which is not observed in TEN or MPE (126). A larger fraction of Tregs is likely to have a more potent ability to migrate into the skin (126). Indeed, increased FOXP3+ T cells in skin lesions of DRESS/DIHS were observed and appear significantly higher when compared to those in SJS/TEN (32, 128). Evidence suggests that functional Tregs expand at the acute stage of DRESS/DIHS and contract with the resolution of the disease while becoming functionally impaired (126). Intriguingly, a shift from the Treg response to the Th17 response due to CD16+ monocytes producing IL-6 can partly explain the observed decrease in Tregs upon resolution (129). Furthermore, Tregs seem important in ameliorating inflammatory responses while preventing the subsequent development of CMV infection or autoimmune diseases in DRESS/DIHS (11).

#### Contact Hypersensitivity

The modulatory role of Treg has also been demonstrated in CHS. *In vivo* Treg expansion in mice produces prolonged CHS suppression manifesting as a sustained reduction of hapten-specific CD8+ T cells and a decrease in effector cell influx in inflamed tissue (130). CD4+ T cells isolated from the peripheral blood of healthy nonallergic humans show a limited capacity to proliferate in response to nickel *in vitro*, but responsiveness is strongly augmented when cells are depleted of Tregs (131).

Although insufficient regulation by Tregs, whether quantitative or qualitative, appears relevant in DTH, the exact mechanisms leading to the pathogenesis of these diseases are still nascent. In the next sections, we attempt to highlight the currently proposed roles of Treg in DTH.

### How Tregs Regulate the Immune Response

**Figure 4:**
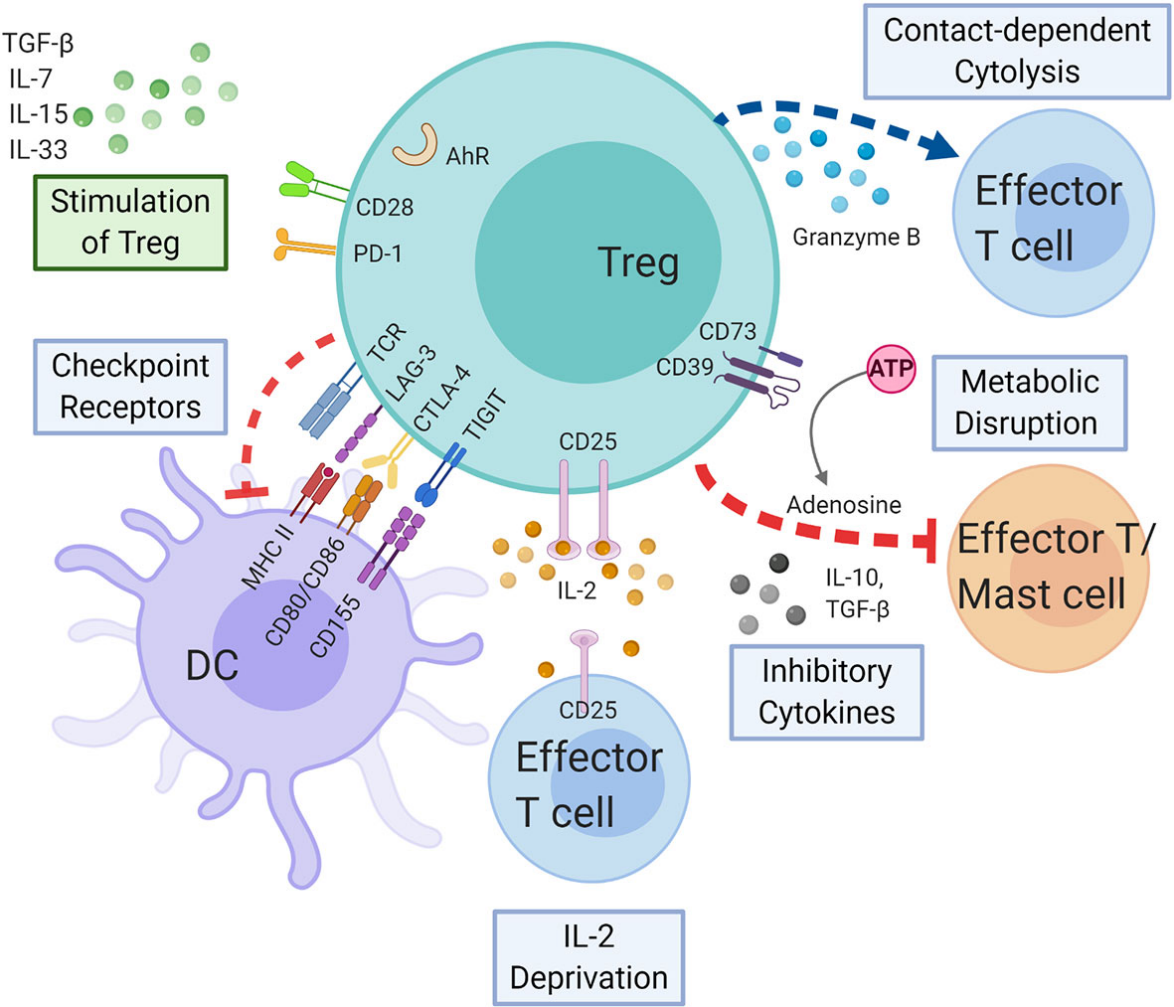
Regulatory T cells in delayed-type hypersensitivity. Tregs may exert immunosuppressive effect on APCs, effector T cells, and mast cells by the following mechanisms: coinhibitory receptors binding to cognate molecules on dendritic cells, secretion of inhibitory cytokines, e.g., IL-10 and TGF-β, metabolic disruption by depriving IL-2 binding and increasing adenosine binding to effector T cells, and contact-dependent cytolysis by granzyme B secretion. Likewise, cytokines and immunoregulatory molecules also modulate the Treg function. AHR, aryl hydrocarbon receptor; ATP, adenosine triphosphate; CTLA-4, cytotoxic T-lymphocyte-associated antigen 4; DC, dendritic cell; IL-2, interleukin 2; IL-7, interleukin 7; IL-10, interleukin 10; IL-15, interleukin 15; IL-33, interleukin 33; LAG-3, lymphocyte-activation gene 3; MHC, major histocompatibility complex; PD-1, programmed cell death protein 1; TCR, T cell receptor; TGF-β, transforming growth factor beta; TIGIT, T cell immunoreceptor with Ig and ITIM domains; Treg, regulatory T cell.

Tregs can regulate participants of the immune system to achieve tolerance through a variety of mechanisms. These mechanisms include suppression by inhibitory cytokines, suppression by metabolic interruption, suppression by checkpoint receptor modulation of DC maturation or function, and suppression by cytolysis (**Figure 4**) (132). Aside from these mechanisms, a novel mechanism involving enhanced binding of Tregs to DC to deplete peptide-MHC II from the surface has been proposed recently (133).

#### Cytokines—Interleukin-10 and Transforming Growth Factor-β

The suppression of various cells, including CD4+ T cells, by Tregs is mediated through inhibitory cytokines including TGF-β, IL-10, and IL-35 (134–137). An early study of mice with disrupted IL-10 gene mounted an exaggerated CHS response (138). Another murine model has demonstrated IL-10 production by Treg upon stimulation and that addition of anti-IL-10 antibodies abrogated the suppressive effects of Treg in CHS. Moreover, CD4+CD25+ T cells isolated from IL-10−/− mice were unable to suppress the immune response (139). In a study of humans with drug hypersensitivity, IL-10 was increased in successful drug desensitization regardless of the hypersensitivity reaction type (140). In patients with MPE, the frequency of IL‐10–producing Tregs was significantly lower compared to controls in both acute and resolution phases (141). Interestingly, the source of IL-10 appears to be important to have suppressive functions. In mice, CD8+ T cell‐derived IL‐10 does not contribute significantly to the resolution of CHS responses (142). One study treated CHS mice with induced Tregs (iTregs) that had been exposed to TGF-β1 small interfering RNA (siRNA) or control siRNA. Mast cell density and inflammatory cytokines messenger RNA expression suppression was compromised in the TGF-β1 siRNA-treated group, suggesting that TGF-β1 derived from iTreg cells might contribute to iTreg–mediated reversal of established CHS (143).

#### Metabolic Interruption: CD39/Adenosinergic Axis and IL-2 Deprivation

CD39 is essential for Treg immunosuppressive activity *via* the CD39/adenosinergic axis since adenosine generated by CD39 and CD73 on Tregs can bind the A2A adenosine receptors on effector T cells and enhance intracellular cAMP levels to suppress their function (144, 145). A murine CHS model has found that CD39 on Tregs depleted ATP in the extracellular environment, which downregulated ATP-induced shedding of CD62L on CD8+ T cells. This in effect interfered with the trafficking of CD8+ T cells in and out of skin-draining lymph nodes and dampened the immune response. Therefore, Tregs likely suppress CHS through a CD39, adenosine-dependent mechanism *in vivo* (146, 147). Higher frequencies of CD39+ Tregs have also been correlated with lower production of IFN-γ, an important cytokine detected in SJS/TEN blisters (121, 148). Furthermore, individuals originally unresponsive to carbamazepine re-exposure converted to an IFN-γ-producing state after treatment with the CD39 inhibitor, POM-1 (121). These data suggest that targeting the CD39/adenosinergic axis has therapeutic potential in SJS/TEN and CHS.

Interestingly, Tregs consistently express high levels of IL-2 receptor α chain, CD25, which implies a higher affinity to IL-2 than that of conventional T cells. By competing for IL-2, Tregs deplete IL-2 from other T cells, causing cytokine deprivation-induced apoptosis in these cells; however, this concept may require further validation (149, 150).

#### Checkpoint Receptors: CTLA-4, PD-1, LAG-3, and TIGIT

Downstream signaling of checkpoint receptors or coinhibitory receptors on Tregs has been shown to converge to the same pathway leading to Treg stability. Furthermore, the coinhibitory receptors may regulate immune and non-immune cells that express their corresponding ligands (151). CTLA-4 in conventional T cells rises only after activation but is constitutively expressed on Tregs (144). Treg-specific CTLA-4 deficiency shows impaired Treg suppressive function, especially in *in vitro* and *in vivo* downregulation of CD80 and CD86 expression on dendritic cells (152). Furthermore, CTLA-4 has been shown to bind to CD80/CD86 with higher affinity than its opponent CD28, thereby depriving conventional T cells of costimulatory signaling through CD28 (153). Tregs may also secrete soluble CTLA-4 that block CD80/CD86 or deplete CD80/CD86 from the APC surface by transendocytosis (154, 155). Additionally, Tregs use CTLA-4 to induce the co-inhibitory molecule PD-L2 (one of the ligand for PD-1) expression on dendritic cells (156).

PD-1 is mainly expressed on activated CD4+ T cells, activated CD8+ T cells, and B cells in the periphery. Just like CTLA-4, PD-1 delivers a negative signal when interacting with its ligands (144). Both PD-1 and PD-1 ligand 1 (PD-L1) are expressed on Tregs under resting and activated conditions (157). As for chronic viral infection, either PD-1 blockade on chronic Tregs or PD-L1 deficiency on CD8+ T cells dramatically diminishes the suppression of T cell immune response, demonstrating the importance of interaction between PD-1 on Tregs and PD-L1 on CD8+ T cells (158). Increasing cases of SJS/TEN secondary to immune checkpoint inhibitors of CTLA-4 and PD-1/PD-L1 suggests checkpoint receptors’ roles in suppressing DTH (159).

The coinhibitory receptor LAG-3 (lymphocyte activation gene-3) is a CD4-related protein that has been demonstrated to modulate both the *in vitro* and *in vivo* suppressive function of induced Tregs (160). MHC II cross-linking by LAG-3 on Tregs induces an inhibitory signaling pathway to suppress dendritic cell maturation and immunostimulatory capacity on conventional T cells (161). Of note, rather opposing functions of LAG-3 have been postulated in a study of DTH in primates: depleting LAG-3+ T cells may eliminate effector T cells to block inflammation but prevent Treg inhibition of immune responses in the draining lymph node (162). Whether LAG-3 plays a role in modulating Tregs’ suppressive function during DTH requires further elucidation.

TIGIT is another coinhibitory receptor that has been shown to contribute to Treg’s suppressive function (163). One of its corresponding ligands CD155 is expressed by dendritic cells and activated CD4+ effector T cells (68, 164). Though both TIGIT and its costimulatory counterpart CD226 bind to CD155, TIGIT binds its ligands with much higher affinity (164, 165). In a murine model, Treg has been suggested to inhibit keyhole limpet hemocyanin-induced delayed-type hypersensitivity reactions by inducing tolerogenic DC through the TIGIT-CD155 interaction (166), which we will discuss later in detail.

#### Cytolysis: Granzyme B

Tregs can also produce a serine protease called GzmB, which can induce apoptosis in effector T cells in a contact-dependent, perforin-independent manner (167). In a murine CHS model, single-gene profiling identified an IL-10/GzmB-expressing Treg population in the draining lymph node with skin-tropic chemokine receptors, which allowed retention of Tregs in inflamed skin and downregulation of the cutaneous immune response (168). However, GzmB is more widely known for its pathogenic role in DTH. The levels of GzmB, along with TNF-α, perforin, and Fas ligand, are markedly increased in PBMCs and blister fluids of SJS/TEN patients (28). GzmB is released to the cytosol of target cells upon cytotoxic T lymphocyte degranulation in SJS/TEN and promotes apoptosis either by direct cleavage of caspase-3 or by increasing the permeability of the mitochondrial outer membrane (intrinsic pathway) (15). Therefore, evidence suggests that GzmB molecules could either regulate or enhance DTH responses depending on their source, such that more caution should be paid when interpreting their role in DTH.

### Regulating the Tregs

While FOXP3+ regulatory T cells have been described as the most physiologically relevant regulatory cell type (144), how other cells and molecules of the immune system regulate Treg development and function to maintain immune homeostasis may be equally important (**Figure 4**). Besides direct cell-cell contact through receptor-ligand interaction, as seen between dendritic cells and T cells, indirect interaction through cytokines also plays important roles in tolerogenic responses of Tregs (169, 170).

#### CD28—CD80/CD86

The CD28-CD80/CD86 interaction on T cells provides a second signal alongside the TCR ligation (signal 1) critical for regulatory T cell survival and the maintenance of immune homeostasis (48). In mice, Treg-specific deletion of CD28 demonstrates lower levels of CTLA-4, PD-1, and chemokine receptor 6 (CCR6) and systemic autoimmunity characterized by prominent skin inflammation (171). In a follow-up study, the upregulation of CCR6 upon CD28 costimulation suggests its role in Treg skin-homing in DTH (172). Interestingly, the percentage of CCR6+ cells among CD4+ T cells has been observed to be greater in SJS/TEN skin than in DRESS/DIHS skin (32).

#### CD25—IL-2

IL-2 is required for sustaining the Treg population to maintain immune homeostasis and tolerance. Since Tregs scarcely produce IL-2, they are highly dependent on exogenous (paracrine) IL-2 for survival (173). As mentioned previously, Tregs express consistently high levels CD25 which serves as a “cytokine sink” to deplete IL-2 from other T cells (149, 150). In a murine model for CHS, IL-2 was required for CD4+CD25+ T cell restriction of the development of the hapten-reactive effector CD8+ T cells (174). Interestingly, by expanding allergen-specific Tregs and reducing pro-inflammatory effector T cells, microparticles (engineered to release TGF-β1, rapamycin, and IL-2) can inhibit hypersensitivity responses to subsequent allergen exposure in an allergen-specific manner, effectively preventing or reversing allergic contact dermatitis in previously sensitized mice (175). While low-dose IL-2 therapy has been recently suggested to have clinical efficacy in a phase I–IIa trial in patients with different autoimmune diseases (176, 177), its therapeutic potential in contact or drug hypersensitivity remains unexplored.

#### Transforming Growth Factor-β

The transforming growth factor-β (TGF-β) family consists of pleiotropic cytokines with both proinflammatory and anti-inflammatory effects, contributing to immune system homeostasis (178). Regarding its connection to Tregs, different studies involving the *Tgfb1*−/− mice have disclosed a multiorgan autoimmunity phenotype and reduced the number of Tregs and expression of FOXP3 (179, 180). Conversion of CD4+ T cells into iTregs or peripheral Tregs (pTreg) is also dependent on TGF-β (181, 182). In mice, the lack of intact TGF-β signaling *via* Smad3 results in an increased proinflammatory, Th2, and Th17 type response in the skin and gastrointestinal tract (183, 184).

#### T Helper 17 Cells

Th17s are a subtype of helper T cells known to cause autoimmunity and inflammation (185). Interestingly, Th17s and iTregs both depend on TGF-β for development. TGF-β can drive Th17 differentiation in the presence of other cytokines, such as IL-6 and IL-21 (186). Higher production of IL-17 in CD4+ T cells expanded from SJS/TEN lesions than those from DRESS/DIHS lesions suggests a role of the Th17/Treg axis in SJS/TEN (32). The percentages of Th17 also tend to be high in SJS/TEN (2–6 days after onset) as compared to normal subjects and MPE patients (187).

#### Aryl Hydrocarbon Receptor

Accumulating evidence indicates that aryl hydrocarbon receptor (AhR) signaling has a specific impact on the generation of Treg. Naïve T cells isolated from AhR null mice inefficiently generate Tregs *in vitro* (188). The absence of AhR signaling leads to heightened inflammation and exacerbated skin pathology in a model of DTH skin reactions in AhR-deficient mice (189). It has been suggested that AhR ligands can regulate the differentiation of Tregs versus Th17 cells by expressing different microRNA signature profile in a model of DTH (190). In humans, the addition of an AhR ligand to naïve T cells differentiated in the presence of TGF-β induces suppressive FOXP3+ Tregs. Furthermore, AhR activation promotes the expression of CD39, an ectonucleotidase that hydrolyzes ATP and mediates the suppressive activity of Treg (191).

#### IL-7

It appears that the regulatory effect of IL-7 is prominent on specialized subpopulations of Tregs rather than on the whole cell lineage. Therefore, contradictory results have been presented regarding the effects of IL-7 on Tregs in the periphery (170). Interestingly, in a murine skin inflammation model, high CD127 (IL-7 receptor α chain) expression correlated with Treg activation. Furthermore, two groups have demonstrated the critical role of IL-7 in the survival of CD127hi Tregs in the skin (192, 193). On the other hand, the percentage of CD4+CD25+CD127- cells, likely Treg cells, increased in T cells from DRESS/DIHS skin lesions compared with those from SJS/TEN (32). Thus, the role of IL-7 in Tregs may differ depending on the type of cutaneous adverse drug reaction and require further exploration.

#### IL-15

Optimal Treg differentiation requires sensing of IL-2, with some compensatory contributions from IL-15 (194). A unique population of CD25-FOXP3+ T cells appears to depend on IL-15 for survival and maturation into Tregs in mice (195). Tregs in human skin proliferate when cultured in contact with dermal fibroblasts and IL-15 (196). However, our recent work on IL-15 has not only linked its elevation in serum to disease severity of SJS/TEN but also demonstrated its ability to induce the production of pathogenic TNF-α, granulysin and GzmB (197). Since IL-15 is vital for maintaining functions of APCs, memory T cells as well as NK cells (198–200), it may not be surprising that antagonizing against IL-15 has been found to inhibit DTH (201). Provided with the multifaceted influences of IL-15 on DTH, more research is warranted to approach the net effect of IL-15 under different circumstances.

#### IL-33

IL-33 is an IL-1-like cytokine that is constitutively expressed by epithelial cells at barrier sites, which functions as an alarmin that is released in response to tissue damage. In a model of murine CHS, disruption of the epidermal barrier induced Tregs *via* IL-33 (202). Although its precise role is yet to be elucidated, serum IL-33 elevation has been observed in SJS/TEN (203).

#### Costimulatory Receptors: ICOS, CD27, and OX40

The core of the CD28 gene family is composed of CD28, CTLA-4, and inducible T cell costimulator (ICOS) (204). ICOS is the most consistent marker for Treg activation which promotes the survival and improves the suppressive function of Tregs (193, 205). In a murine model of CHS, a population of CD4+CD25+FOXP3+ T cells upregulated ICOS on *in vivo* sensitization and specifically suppressed hapten-reactive CD8+ T cells both *in vivo* and *in vitro* (206). Langerhans cells also seem to prevent the development of CHS by activating ICOS+CD4+FOXP3+ Tregs (207). Intriguingly, two TNFRSF members, CD27 and OX40, are preferentially expressed by skin-resident Tregs. Both CD27 and OX40 signaling suppress the expression of Th17-associated genes from Tregs *in vitro* and *in vivo*. Tregs that lack both CD27 and OX40 are compromised in terms of controlling skin inflammation and expressed high levels of IL-17A (208). Therefore, the potential roles of CD27 and OX40 on Tregs and the Treg/Th17 axis in SCARs may deserve future investigation.

## Coinhibitory Receptors

Besides the inhibitory Treg, various coinhibitory receptors also prevent T cells from being activated under different conditions. Thus, coinhibitory receptors involved in hypersensitivity immune responses also deserve detailed investigation to identify potential druggable targets (**Table 2**).

**Table 2:** Coinhibitory molecules involved in delayed-type hypersensitivity.

Coinhibitory molecule	Implicated cells in DTH	Ligand/receptor	Ligand/receptor expression	Type of DTH	Study designs
CTLA-4	Tregs, activated T cells	CD80, CD86	APCs	CHS; irAE; efavirenz and NSAID hypersensitivity; SMX-NO-induced hypersensitivity; delayed type liver injury to isoniazid, nevirapine, and amodiaquine	retrospective cohort study (209); case control (210, 211); case series (119, 212); case report (213); animal study (214–217); *in vitro* study (124)
PD-1	Tregs, activated T cells, CD8+ Trm cells	PD-L1	Tregs, conventional T cells, APCs	CHS; irAE; SMX-NO-induced hypersensitivity; delayed type liver injury to isoniazid, nevirapine, and amodiaquine	Retrospective cohort (209) study; case series (119); case report (123, 213, 218); animal study (215–217, 219); *in vitro* study (114, 124)
TIM-3	Tregs, activated T cells, CD8+ Trm cells	Galectin 9	APCs	SMX-NO-induced hypersensitivity; MPE; CHS	Case control (141); animal study (219); *in vitro* study (124)
LAG-3	Activated T cells, Tregs	MHC II	APCs	Tuberculin-induced DTH	Animal study (162)
TIGIT	T cells (including Tregs), NK cells	CD155	APCs, T cells	Keyhole limpet hemocyanin-induced DTH	Animal study (166)
BTLA	T cells	HVEM	APCs, T cells, NK cells	CHS	Animal study (220)

DTH, delayed type hypersensitivity; CTLA-4, cytotoxic T lymphocyte-associated antigen-4; Treg, regulatory T cells; APC, antigen-presenting cell; CHS, contact hypersensitivity; irAE, immune-related adverse events; NSAID, non-steroid anti-inflammatory drugs; SMX-NO, nitroso sulfamethoxazole; PD-1, programmed cell death 1; Trm cells, tissue-resident memory T cells; TIM-3, T-cell immunoglobulin mucin-3; MPE, maculopapular exanthema; LAG-3, lymphocyte activation gene-3; MHC, major histocompatibility complex; TIGIT, T cell immunoglobulin and ITIM domain; BTLA, B and T lymphocyte attenuator; HVEM, herpesvirus-entry mediator.

### CTLA-4 and PD-1

The most well-known coinhibitory receptors are perhaps CTLA-4 and PD-1 due to the application of checkpoint blockade in cancer immunotherapy (221, 222). CTLA-4 and CD28 are homologous receptors expressed on both CD4+ and CD8+ T cells that bind to CD80/CD86 on APCs; but unlike CD28, CTLA-4 serves to ultimately inhibit T-cell responses (223, 224). There have been several proposed cell-intrinsic and cell-extrinsic immunosuppressive mechanisms of CTLA-4, which suppress the cell carrying CTLA-4 or the cell carrying CD80/CD86, respectively (225). Thus, enhancing CTLA-4 activity may be beneficial in attenuating unwanted immune responses. Regarding the role of CTLA-4 in DTH, one murine study has found that CTLA-4-Immunoglobulin (Ig) provided long-term immunosuppression against both DNFB- and oxazolone-induced CHS in a dose-dependent manner. Inhibited activation of T cells in the draining lymph node and maturation of DCs and B cells, reduced infiltration of activated CD8+ T cells into inflamed tissue, and decreased cytokines and acute-phase proteins at the inflamed tissue and in circulation demonstrate the immunosuppressive function of CTLA-4-Ig both locally and systemically (214). The CTLA-4–Ig fusion protein, abatacept, has shown promise in treating rheumatoid arthritis in humans (226), while its use in various autoimmune diseases is being tested in clinical trials (227).

Not surprisingly, CTLA-4 mutations or polymorphisms have been observed in patients with autoimmune diseases and patients with Efavirenz and nonsteroidal anti-inflammatory drug hypersensitivity (210, 211, 228, 229), suggesting CTLA-4　alteration may disrupt homeostatic function, resulting in the observed disease phenotype. Indeed, deprivation of CTLA-4 leads to an enhanced immune response to drugs, as seen in the following studies. PD-1 knockout mice treated with amodiaquine and anti-CTLA-4 demonstrated liver injury similar to idiosyncratic drug-induced liver injury (IDILI) in humans with increased portal infiltration of lymphocytes and perforin and granzyme (215). Idiosyncratic drug reaction to isoniazid and nevirapine manifesting as delayed-type liver injury has been also been suggested by this model of PD-1 knockout mice treated with anti-CTLA-4 (216, 217). In the presence of CTLA-4 blocking antibodies, enhanced naïve T cell proliferative response and memory drug antigen-specific responses to SMX-NO are observed in an *in vitro* assay (124).

PD-1, as mentioned previously, is a checkpoint receptor mainly expressed on activated CD4+ T cells, activated CD8+ T cells, and B cells in the periphery, and it similarly transmits an immunosuppressive signal during ligation, inhibiting the proliferation, cytokine generation and release, and cytotoxicity of T cells (144, 230). Its ligand PD-L1 is expressed on T cells, B cells, DCs, macrophages, and non-hematopoietic cells, while another of its ligand, PD-L2, is expressed mainly on APCs (231). PD-1 is known to regulate T cell effector functions during various physiological responses, including acute and chronic infection, cancer, autoimmunity, and in immune homeostasis (232). In fact, agonist antibodies to PD-1 and other coinhibitory receptors have demonstrated promise in treating animal models of autoimmune diseases (233).

The blockade of the PD-1/PD-L1 axis has also been demonstrated to play a role in contact and drug hypersensitivity. In a murine study using the experimental model of CHS to DNFB, epidermal CD8+ tissue-resident memory T (Trm) cells expressing PD-1 seem to ameliorate CHS. Blocking PD-1 *in vitro* with anti–PD-1 monoclonal antibodies increased the reactivity of epidermal CD8+ Trm cells and their capacity to produce IFN-γ and GzmB on *ex vivo* reactivation. Furthermore, when animals with DNFB allergy were depleted of circulating CD8+ T cells, blockade of PD-1 triggered severe flare-up CHS reactions after low-dose DNFB re-exposure (219). Another study using PBMCs from human donors demonstrated that priming of drug-naive CD4+ and CD8+ T cells against drug antigens was more effective when PD-L1 signaling was blocked (114). However, memory T cell responses to drug antigens were not similarly enhanced by PD-L1 blockade (124).

We previously discussed that checkpoint receptor blockade may lead to disequilibrium of the immune system manifesting as boosted T cell response. In a case series of four melanoma patients, increased risk of sulfasalazine-induced cutaneous hypersensitivity was observed after treatment with the anti-PD-1 inhibitor pembrolizumab and/or the anti-CTLA-4 inhibitor ipilimumab (119). One patient developed fever with erythematous maculopapular rash and liver injury while another developed fever with unspecified rash and facial edema, though skin biopsy was not obtained for suspected SCAR. In another recent study, one patient developed TEN one month after discontinuing nivolumab. Her lymphocyte stimulation tests were simultaneously positive for several concomitant drugs while elevated levels of autoantibodies were noted (123). These observations suggest that checkpoint inhibitors may render patients more vulnerable to developing hypersensitivity to other drugs and autoimmunity. On the other hand, checkpoint inhibitors, though quite successful in treating various cancers, have autoinflammatory side effects termed immune-related adverse events (irAEs), including the commonly seen cutaneous toxicities (234). Although pruritus and MPE are more common, severe forms of cutaneous manifestations such as SJS/TEN, DRESS/DIHS, and Sweet’s Syndrome can occur on rare occasions (209, 218, 235, 236). In a melanoma patient treated with ipilimumab and nivolumab, morbilliform eruption progressed to TEN over 3 months. During the course of treatment, an increase of CD8+ T cells within the dermal-epidermal junction and an increase of PD-L1 expression in both T cells and keratinocytes were noted on skin biopsy (213). Similarly, one case series reported that 22% of the 68 patients on pembrolizumab or nivolumab developed inflammatory skin lesions ranging from mild maculopapular rash to SJS-like lesions with expression of PD-1 on skin-infiltrating T cells and keratinocytes by immunohistochemistry. Further gene expression analysis of lesional skin revealed a profile resembling SJS/TEN (212). Together, these data suggest that proper PD-1/PD-L1 functions are important in maintaining epidermal integrity while T-cell, antibody, and cytokine responses are likely involved in irAE pathogenesis (237). Although the complex mechanism of severe cutaneous reactions in patients using checkpoint inhibitors has yet to be revealed, the pivotal role of CTLA-4 and PD-1/PD-L1 in their pathogenesis has been sufficiently demonstrated.

### TIM-3

In addition to CTLA-4 and PD-1, “second-generation” coinhibitory receptors and ligands that belong to the B7 family are emerging as potential new targets in cancer immunotherapy (238). One of these receptors is T cell immunoglobulin-3 (TIM-3). TIM-3 mediates the T cell function through binding to galectin-9 (Gal-9), leading to the death of predominantly Th1-specific T-cells, which has been shown to be defective in patients with drug‐induced MPE (124, 141). These patients exhibited reduced levels of TIM-3 in Th1 cells and impaired expression of Gal-9 in PBMCs and dendritic cells, while the addition of exogenous Gal-9 significantly reduced Tim3+ Th1 proliferation. However, another study has found that blocking TIM-3 did not enhance the proliferative response of SMX-NO-primed naïve or memory T-cells from any human donors (124). In a murine model of CHS, *in vitro* TIM-3 blockade, similar to PD-1 blockade, increased the activity of epidermal CD8+ Trm cell activity and cytokine production, though the effect was more modest compared to that of PD-1 (219). It appears that the effect of blocking TIM-3 variably dampened the immune response depending on the type of DTH and the study species. Therefore, the net effect of TIM-3 on DTH in the interplay of different co-signaling receptors is still warranted, especially in the insufficiently explored area of SCARs.

### LAG-3

Lymphocyte activation gene-3 (LAG-3) is expressed on activated CD4+ and CD8+ T cells, natural killer cells, and myeloid cells (239). A recent study has suggested that LAG-3 preferentially suppresses T cells responsive to stable complexes of peptide and MHC class II by transducing inhibitory signals *via* its intracellular region (240). LAG-3 suppresses T cell activation and cytokine secretion and seems to work synergistically with PD-1 to inhibit immune responses, making anti-LAG-3 an important player in cancer immunotherapy (241). A study has demonstrated that depleting activated T cells with a LAG-3 cytotoxic antibody prevented T cell-driven skin inflammation in a preclinical DTH model in non-human primates (162). The result from this study highlights that antibody-mediated depletion of LAG-3-activated T cells might have therapeutic potential in DTH, which deserves future validation in humans.

### TIGIT

TIGIT a coinhibitory receptor highly expressed on Tregs, as mentioned previously in section 4.2.3. However, it is also expressed on other T cells and even NK cells (166). TIGIT expression varies with different diseases, and its expression has been shown to be upregulated in T cells and causes dysfunction independent of PD-1 and TIM-3 in leukemic patients (242, 243). While its potential as a therapeutic target in cancer is being explored, its role in DTH remains elusive. One murine study previously demonstrated that less ear swelling was seen in keyhole limpit hemocyanin-(KLH-) treated mice immunized with the TIGIT-Fc or CTLA-4–Fc fusion protein. However, TIGIT-Fc’s inhibition of DTH was not observed in IL-10 knockout mice, suggesting that IL-10 is involved in TIGIT function (166). Results from this study suggested the differential regulatory mechanisms of each coinhibitory receptor.

### BTLA

B and T lymphocyte attenuator (BTLA) is a coinhibitory receptor which belongs to the Ig superfamily and resembles PD-1 and CTLA-4 (244). BTLA is expressed on CD4+ and CD8+ T cells, B cells, and other APCs (245). The ligation of BTLA by the herpesvirus-entry mediator (HVEM) on APCs attenuates T cell proliferation (246). BTLA signaling pathway is also defective in T cells in autoimmune diseases, such as systemic lupus erythematosus (247). Regarding its potential role in CHS, BTLA−/− mice display enhanced DNFB-induced CHS and CD8+ T cell proliferation and IFN-γ production compared with the wild-type mice. Consequently, *in vivo* injection of agonist anti-BTLA antibody is able to suppress DNFB-induced CHS and IFN-γ production, suggesting that stimulation of BTLA has therapeutic potential in CHS (220). However, in the previously mentioned study by Gamradt et al. unlike PD-1 and TIM-3, BTLA expression was not detected in epidermal CD8+ Trm cells in mice with CHS (219). Therefore, the expression profile of BTLA deserves further investigation to verify its potential role in regulating the immune response to drug and contact antigen.

## Conclusion

DTH manifesting as SJS/TEN and DRESS/DIHS are potentially lethal due to the involvement of internal organs, generating an overwhelming systemic inflammatory storm in addition to affecting the skin. It is therefore imperative to preceisly understand the complex pathogenesis of SCARs for appropriate treatment. As we have outlined in this review, a variety of costimulatory and coinhibitory receptors have been suggested by different studies to play a role in DTH. Unfortunately, the caveat is that research in this area is still in its early stage, such that much of what is currently known about co-signaling receptors in DTH is largely based on murine models, *in vitro* studies, or small retrospective studies. Additionally, while individual contributions of each co-signaling receptor or Treg has been suggested, the interplay between them in the formation of the disease phenotype is largely unexplored. All in all, future research should embrace a multi-faceted approach in order to explore the interplay between a variety of co-signaling pathways while also aim at clinical-based studies since animal models may not fully represent the pathogenesis in human.

## Author Contributions

Y-SH, K-LL, YF, W-HC, and C-BC conceptualized the review. W-HC and C-BC provided the resources. Y-SH, K-LL, YF, C-WW, C-WL, Y-FL, W-HC, and C-BC wrote the original draft. K-YY, W-CC, S-IH, W-HC, and C-BC reviewed and edited the draft. Y-SH, K-LL, and C-BC created the visualizations. W-HC and C-BC supervised. C-BC coordinated the project administration. W-HC and C-BC acquired funding. All authors contributed to the article and approved the submitted version.

## Funding

This study was supported by research grants from the Ministry of Science and Technology, Taiwan (MOST 108-2314-B-182A-006-MY3 to CB Chen; MOST 103-2321-B-182-001, MOST 104-2314-B-182A-148-MY3, MOST 104-2325-B-182A-006, MOST 105-2325-B-182A-007, MOST 106-2314-B-182A-037-MY3, MOST 106-2622-B-182A-003-CC2, MOST 107-2622-B-182A-001-CC2, MOST 108-23 14-B-182A-104-MY3, MOST 108-2320-B-182A-023-MY3, MOST 108-2320-B-182A-024-MY2, MOST 109-2320-B-182A-008 -MY3, MOST-108-2314-B-182A-104 -MY3, MOST-109-2326-B-182A-001 to WH Chung), and Chang Gung Memorial Hospital, Taiwan (CMRPG2H0081, CMRPG2J0221, CMRPG2J0222, NMRPG2J6012, NMRPG2J6013, CIRPG2I0011, CIRPG2I0012, CIRPG2I0013 to CB Chen; CIRPG3I0022, CIRPG3I0023, CIRPG3I0042, CIRPG3I0043, CLRPG3E0036, CLRPG3J0012, CMRPG3I0382, CORPG3J0322, CLRPG2E0053, CMRPG3D0363, CORPG3F0042~3, NCRPG3G0023, NCRPG3GS023, NMRPG3G6293, NMRPG3J6062, NMRPG3J6063, NMRPG3K0521, OMRPG3E0041 to WH Chung).

## Conflict of Interest

The authors declare that the research was conducted in the absence of any commercial or financial relationships that could be construed as a potential conflict of interest.

## Abbreviations

ACD, allergic contact dermatitis; APC, antigen-presenting cell; BTLA, B and T lymphocyte attenuator; CCR6, chemokine receptor 6; CHS, contact hypersensitivity; CMV, cytomegalovirus; CTL, cytotoxic T lymphocyte; CTLA-4, cytotoxic T lymphocyte-associated antigen-4; DC, dendritic cell; DIHS, drug-induced hypersensitivity syndrome; DNFB, dinitrofluorobenzene; DRESS, drug reaction with eosinophilia and systemic symptoms; DTH, delayed-type hypersensitivity; EBV, Epstein-Barr virus; FOXP3, Forkhead protein 3; Gal-9, galectin-9; GITR, glucocorticoid induced TNF receptor; GzmB, granzyme B; HHV, human herpes virus; HIV, human immunodeficiency virus; HLA, human leukocyte antigen; HVEM, herpesvirus-entry mediator; ICOS, inducible T cell costimulator; IFN, interferon; IL, interleukin; IPEX, immunodysregulation, polyendocrinopathy, enteropathy, X-linked; IrAE, immune-related adverse event; LAG-3, lymphocyte activation gene-3; MHC, Major histocompatibility complex; MPE, maculopapular exanthema; NK, natural killer; PBMC, peripheral blood mononuclear cell; PD-1, programmed cell death 1; SCAR, severe cutaneous adverse reaction; SJS, Stevens-Johnson syndrome; SMX, sulfamethoxazole; TARC, thymus activation-regulated chemokine; TBSA, total body surface area; TCR, T cell receptor; TEN, toxic epidermal necrolysis; TGF-β, transforming growth factor-beta; Th1, T helper 1 cell; Th17, T helper 17 cell; TIGIT, T cell immunoglobulin and ITIM domain; TIM-3, T-cell immunoglobulin mucin-3; TNF, tumor necrosis factor; TNFRSF, tumor necrosis factor receptor superfamily; Treg, regulatory T cell; Trm, tissue-resident memory T cell.
